# Ultrasound-based multimodal diagnosis and endovascular therapy for phlegmasia cerulea dolens secondary to May–Thurner syndrome in a middle-aged male patient: A case report

**DOI:** 10.1097/MD.0000000000051048

**Published:** 2026-10-09

**Authors:** Weiwei Jiang, Ying Zhang

**Affiliations:** a Department of Ultrasound Medicine, People’s Hospital of Honghuagang District, Zunyi, Guizhou, China.

**Keywords:** case report, deep vein thrombosis, iliac vein compression syndrome, phlegmasia cerulea dolens, ultrasound diagnosis

## Abstract

**Rationale:**

This study explores the value of ultrasound in the diagnosis of patients with phlegmasia cerulea dolens (PCD). The enrolled patient suffered from PCD secondary to iliac vein compression syndrome (IVCS) and complicated by extensive lower-limb deep vein thrombosis (DVT). We analyzed an atypical case in a middle-aged male. We summarized the advantages and limitations of ultrasound. We aimed to provide practical references for multimodal diagnosis and emergency management of critical DVT patients.

**Patient concerns:**

A 54-year-old male patient was diagnosed with IVCS. The condition was complicated by extensive left lower-limb DVT and subsequent PCD. We retrospectively collected his clinical data, imaging findings, and interventional records. We summarized the typical ultrasound features. We combined computed tomography angiography (CTA) and venography results. We further clarified the clinical role of ultrasound in such life-threatening vascular emergencies.

**Diagnoses:**

Based on clinical manifestations and multimodal imaging examinations, the final diagnoses were phlegmasia cerulea dolens (PCD) secondary to iliac vein compression syndrome (IVCS), complicated by extensive left lower-limb deep vein thrombosis.

**Interventions:**

The patient received emergency endovascular interventional therapy, including thrombus aspiration, catheter-directed thrombolysis, and iliac vein stent implantation. Anticoagulant therapy was administered perioperatively. Serial ultrasound examinations were performed before and after intervention to dynamically evaluate venous hemodynamics.

**Outcomes:**

Ultrasound rapidly detected extensive thrombosis in the left femoral vein, popliteal vein, and calf intermuscular veins. It clearly displayed thrombus location, scope, and echogenic features. It provided critical imaging evidence for emergency intervention. However, ultrasound could not directly visualize iliac vein stenosis. Pelvic anatomical depth and intraluminal thrombus obstruction mainly accounted for this limitation. CTA and venography successfully confirmed the underlying IVCS etiology. Postoperative ultrasound hemodynamic parameters objectively evaluated vascular recanalization efficacy. Combined interventional treatment effectively restored limb blood flow. Lower-limb edema and pain were significantly relieved after treatment.

**Lessons:**

Ultrasound serves as the first‑line screening tool for PCD. It allows rapid and real‑time evaluation of thrombus burden in the emergency department. Routine postoperative hemodynamic ultrasound can objectively assess vascular recanalization for therapeutic monitoring. However, ultrasound carries a high risk of missed diagnosis of primary iliac vein stenosis. For highly suspected IVCS cases, CTA and venography are essential for etiological confirmation. A multimodal collaborative diagnosis and treatment strategy is strongly recommended for critical PCD patients.

## 1. Introduction

Iliac vein compression syndrome (IVCS; May–Thurner syndrome) arises from chronic compression of the left common iliac vein by the right common iliac artery and the fifth lumbar vertebra, resulting in endothelial injury, inflammation, and fibrotic stenosis.^[1]^ While classically described in young women, male patients often present with more severe, acute-onset disease that rapidly progresses to extensive deep vein thrombosis (DVT) and phlegmasia cerulea dolens (PCD) – a limb-threatening emergency with mortality rates up to 40%.^[2,3]^ PCD secondary to IVCS poses 3 critical diagnostic challenges: its presentation (acute swelling, cyanosis, and diminished arterial pulses) mimics arterial thrombosis; the disease progresses rapidly, with venous gangrene developing within hours; and bedside color Doppler ultrasound, although highly sensitive for distal thrombus, frequently fails to directly visualize iliac vein stenosis due to pelvic depth, bowel gas, and obscuration by acute intraluminal thrombus.^[4]^ Consequently, a stepwise multimodal imaging strategy – anchored by ultrasound screening and supplemented by computed tomography angiography (CTA) or venography – is essential for timely diagnosis and intervention. Herein, we report a 54-year-old male with IVCS-induced total left lower-limb DVT progressing to PCD and delineate the hierarchical role of ultrasound within this multimodal emergency pathway.

## 2. Case presentation

### 2.1. Medical history

A 54-year-old male presented to the emergency department with sudden swelling and pain in the left lower limb for more than 5 hours. He had no prior thrombotic or chronic diseases but reported long-term smoking and intermittent alcohol consumption. The outpatient department initially misdiagnosed him with acute lower-limb arterial thrombosis and admitted him to the neurology center. Although IVCS classically affects women aged 20 to 40 years, this 54-year-old male exemplifies the acute, high thrombus-burden IVCS-DVT phenotype frequently observed in men. His onset was acute, disease progression was rapid, and PCD developed within a short time.

### 2.2. Physical examination and laboratory findings

Physical examination revealed diffuse swelling of the left lower limb from the iliac crest to the dorsum of the foot. The circumference of the left lower limb was approximately 7 cm and 3 cm longer than that of the right lower limb, respectively, at 15 cm above the knee and 10 cm below the knee. The skin temperature was reduced, and the skin was bluish-purple and tender (+++) (Fig. 1A). There were no palpable pulses in the left dorsalis pedis, anterior or posterior tibial arteries, while normal palpable pulses were detected in the right lower limb. The other vital signs were as follows: temperature 36.7°C, pulse rate 70 beats/min, respiratory rate 20 breaths/min, and blood pressure 87/65 mm Hg, indicating a hypovolemic or shock state. Laboratory findings revealed markedly elevated D-dimer (3.27 mg/L vs reference range < 0.5 mg/L) and coagulation abnormalities with mild prolongation of prothrombin time and activated partial thromboplastin time. Elevated white blood cell (12.06 × 10^9^/L) and neutrophil (7.04 × 10^9^/L) counts suggested an acute inflammatory response and hypercoagulable state.

**Figure 1.**
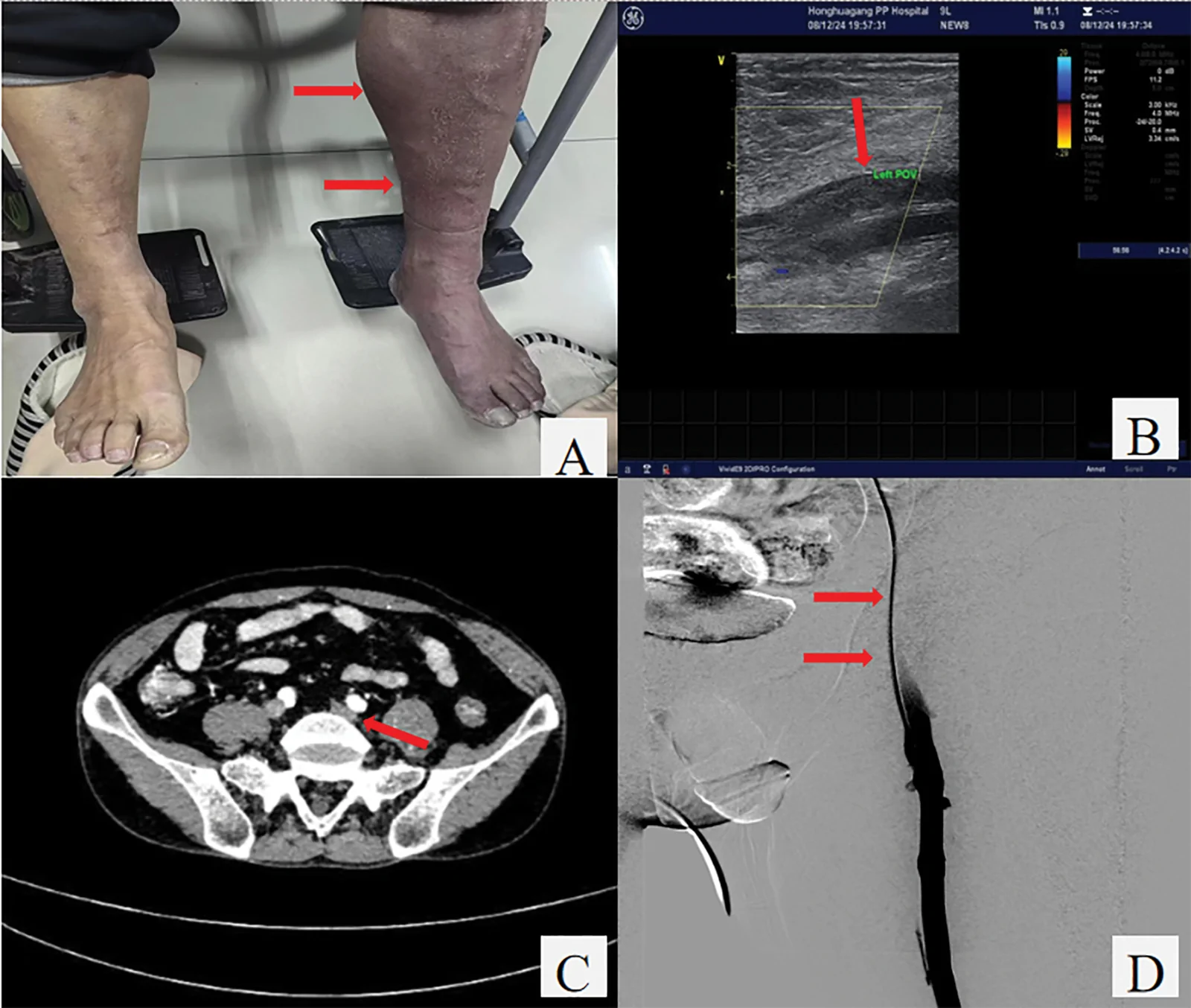
Clinical manifestations and multimodal imaging findings of the patient with phlegmasia cerulea dolens secondary to iliac vein compression syndrome. (A) Photograph of bilateral lower extremities: marked diffuse swelling, cyanotic discoloration, and increased skin tension over the left lower limb (red arrows). (B) Color Doppler ultrasound of the left popliteal vein demonstrates complete intraluminal thrombosis with absent blood-flow signal (red arrow). Complete stored ultrasound panels of the external iliac vein were not prospectively archived during this emergency scan; full-range thrombus involvement is described in textual findings. (C) Axial contrast-enhanced CT of the pelvis shows mechanical compression of the left common iliac vein at its crossing with the right common iliac artery and anterior to the L5 vertebral body (red arrow). Quantitative CTA measurements (see text) revealed mild-to-moderate stenosis (48% diameter reduction, 39% area reduction). (D) Digital subtraction venography: extensive filling defects within the left iliac and proximal femoral veins, consistent with multi-segmental deep vein thrombosis (red arrows). CT = computed tomography, CTA = computed tomography angiography.

### 2.3. Imaging findings

Ultrasound examination revealed extensive thrombosis of the left popliteal, posterior-tibial, and calf intermuscular veins. Thrombus was suspected to extend proximally to the external iliac vein. Vessels were dilated without a compressible lumen. No blood flow or spectral signals could be detected by color Doppler flow imaging and pulsed wave Doppler. In addition, absence of blood flow signals was noted in the anterior tibial artery, posterior tibial artery, and dorsal foot arteries of the left lower limb, suggesting acute secondary arterial involvement.

CTA clearly demonstrated mechanical compression of the left common iliac vein. The site of compression was located at the crossing point with the right common iliac artery and at the anterior margin of the fifth lumbar vertebra (L5) (Fig. 1C; the red arrow indicates the site of compression). Lower-limb CTA measured the inner diameter of the left common iliac vein at its narrowest point at the L5 level to be approximately 4.6 mm, with a luminal area of approximately 76 mm^2^; At the same level, the right common iliac vein had an internal diameter of approximately 8.8 mm and a luminal area of approximately 125 mm^2^. The percentage reduction in diameter was approximately 48% and the percentage reduction in area was approximately 39%, suggesting mild-to-moderate compressive stenosis of the left common iliac vein. Extensive filling defects were observed in the left external iliac vein and all distal deep veins.

Lower-limb venography further confirmed acute thrombosis in the left iliac and femoral veins. Contrast medium stasis was observed in the distal segment of the common femoral vein, with no evidence of significant compensatory collateral circulation (Fig. 1D).

### 2.4. Diagnosis

The patient was ultimately diagnosed with left lower-limb PCD, which was total lower-limb DVT secondary to IVCS. This diagnosis was primarily based on the following:

1. Clinical presentation: sudden swelling, severe pain, cyanosis of the left lower limb (Fig. 1A), and absent dorsalis pedis pulse (corresponding to the PCD triad).
2. Laboratory findings: markedly elevated D-dimer (3.27 mg/L), prolonged prothrombin time, and activated partial thromboplastin time, which indicated acute thrombosis and a hypercoagulable state.
3. Imaging: ultrasound revealed complete thrombosis of the left popliteal vein, posterior tibial vein, and intermuscular veins of the lower leg, with dilated lumina, absence of blood flow signals, and soft tissue edema; the thrombus was suspected to extend superiorly into the external iliac vein (Fig. 1B). CTA revealed mechanical compression of the left common iliac vein and quantitatively confirmed mild-to-moderate compressive stenosis (diameter stenosis rate 48%, cross-sectional area stenosis rate 39%; Fig. 1C). Venography confirmed acute thrombosis of the left iliofemoral vein (Fig. 1D). The combination of multimodal imaging clearly identified IVCS as the underlying cause of the thrombosis and PCD.

### 2.5. Treatment protocol

#### 2.5.1. Timeline of emergency management

Within 0 to 30 minutes after admission, we completed history collection, physical examination, and laboratory tests. High clinical suspicion of left lower-limb DVT and PCD was confirmed. Within 30 to 60 minutes, emergency ultrasound was completed. It confirmed extensive DVT and excluded acute arterial embolism.

Within 60 to 90 minutes, emergency CTA and venography were performed. The combined examination confirmed left IVCS complicated with total left lower-limb DVT. Within 90 to 120 minutes, multidisciplinary discussion was conducted. Combined interventional therapy was determined. The patient signed informed consent.

Within 2 hours after admission, all emergency interventions were successfully completed. The procedures included inferior vena cava filter implantation, left iliac vein balloon dilatation, AngioJet (Boston Scientific, Marlborough) mechanical thrombectomy, and thrombolytic catheter placement. At each time node, clinical decision-making was driven as follows: clinical suspicion of PCD was raised by history and vital-sign instability; emergency ultrasound excluded primary arterial occlusion and confirmed extensive DVT; CTA-venography identified underlying IVCS; a multidisciplinary team discussion balanced risks and benefits of different therapeutic options before endovascular intervention was performed after obtaining written informed consent.

#### 2.5.2. Rationale for the choice of treatment strategy

The combined interventional strategy was formulated based on the patient’s critical condition. First, the patient had developed typical PCD. It represented complete venous outflow obstruction. Simple anticoagulation could not rapidly restore limb perfusion or save the endangered limb. Second, IVCS was the fundamental etiology. Intraoperative angiography confirmed mechanical iliac vein compression. Balloon dilatation was necessary to relieve stenosis and reduce thrombosis recurrence. During the acute phase, balloon dilatation is performed without immediate stent implantation, primarily because a large amount of fresh thrombus obscures the true fibrotic damage to the venous wall; once the thrombus burden has decreased significantly, imaging follow-up can be used to assess whether secondary iliac vein stent implantation is required. The timing for secondary stenting is generally chosen when the thrombus has largely resolved and the vascular wall lesions can be fully assessed, which is usually 1 to 3 months postoperatively. Third, the patient had an extremely high thrombus burden with complete venous occlusion. AngioJet mechanical thrombectomy could rapidly reduce thrombus volume and decrease acute venous hypertension. As the original parameters from the emergency surgery record were lost, no AngioJet pump operating parameters are available for this case; at our center, routine AngioJet mechanical thrombectomy procedures are performed in suction mode, with each suction cycle lasting 48 to 60 seconds and intervals of 3 to 5 minutes, to minimize hemolysis-related complications. Fourth, retrievable inferior vena cava filter implantation was performed to prevent pulmonary embolism secondary to thrombus dislodgement during mechanical thrombectomy and catheter-directed thrombolysis (CDT). Fifth, postoperative CDT cleared residual micro-thrombus. It reduced the incidence of post-thrombotic syndrome (PTS). Simple surgical open thrombectomy was not selected given the patient’s hemodynamically unstable status and high perioperative morbidity. Primary iliac vein stenting was deferred at the acute stage because severe thrombus burden hampered accurate assessment of true venous wall lesion. Therefore, combined endovascular strategy represented the most reasonable option for this life‑threatening PCD patient.

In this case, the postoperative anticoagulation regimen was based on the Chinese guidelines for the diagnosis and treatment of DVT, and rivaroxaban was selected for standardized anticoagulation therapy.

#### 2.5.3. Surgical procedure and key parameters

Bilateral inguinal regions were routinely disinfected and draped. Local anesthesia was performed with 1% lidocaine. The right femoral vein was successfully punctured via the Seldinger technique, and a 5F vascular sheath was inserted. Inferior vena cava angiography showed a smooth vascular lumen and an intact vessel wall. Bilateral renal vein openings were located at the upper edge of the L2 vertebral body. A guidewire was advanced to the superior vena cava. A retrievable vena cava filter sheath was delivered to the L2 upper margin. The filter was accurately deployed 0.5 cm below the renal vein orifices. Repeated angiography confirmed a stable and centered filter position without displacement. Subsequently, left lower-limb venography was performed. A 10F sheath was exchanged. Severe left iliac vein stenosis and thrombosis blocked catheter passage. Under stiff guidewire guidance, the angiographic catheter reached the superficial femoral vein segment. Angiography confirmed severe left iliac vein stenosis and obstructive thrombosis. A 6 mm × 12 mm PTA balloon was delivered to the stenotic segment. Primary dilatation was performed at 12 atm for 3 minutes. The stenosis was slightly improved, but venous return remained sluggish. Secondary dilatation was performed at 14 atm for 3 minutes. Angiography showed significant improvement of iliac vein stenosis. Limb venous outflow was improved, though minor residual thrombus remained. A thrombolytic catheter was placed in the left superficial femoral vein. The operation proceeded smoothly. A large amount of thrombus was retrieved during the procedure (Fig. 2A), and the patient’s vital signs remained stable throughout the operation. A total of 270 mL iopromide was used, and 30 mL was discarded. Immediately after surgery, left limb swelling and pain were significantly relieved, and cold skin sensation resolved. The original AngioJet pump settings and post-procedural CDT infusion rates were unavailable from the emergency surgical log.

**Figure 2.**
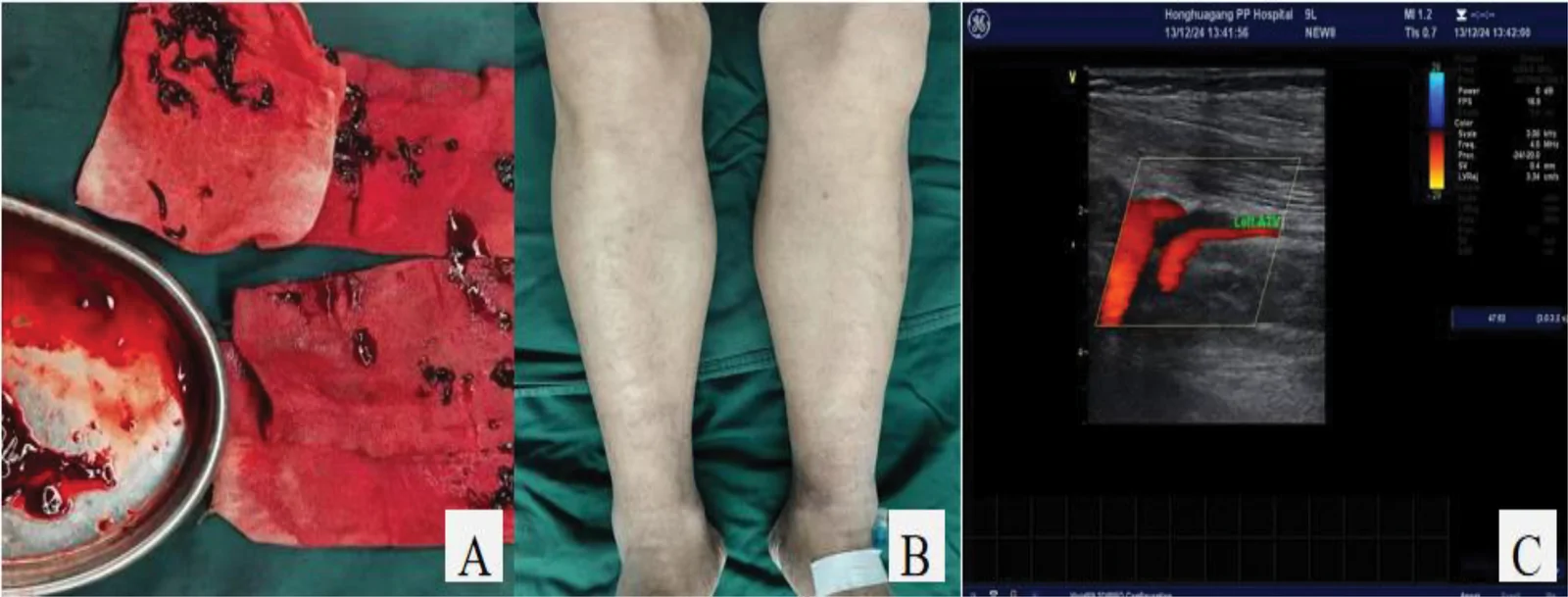
(A) Thrombus specimens retrieved during endovascular thrombectomy. (B) Clinical photograph shows marked improvement in left lower-limb swelling and skin discoloration after intervention. (C) Postoperative color Doppler flow imaging demonstrates restored blood-flow signals within the anterior tibial vein.

### 2.6. Follow-up and outcomes

After 5 days, the patient’s symptoms had significantly improved (Fig. 2B). Lower-limb swelling subsided, with circumference differences reduced to 2 cm above and 1 cm below the knee; skin temperature and color returned to normal, and the dorsalis pedis pulse resumed. These clinical improvements were confirmed by imaging studies. Ultrasound follow-up demonstrated significant recanalization of the deep venous system, with restored blood flow signals in the anterior tibial vein (Fig. 2C) and improved spectral waveforms in the femoral vein.

Quantitative hemodynamic assessment by ultrasound revealed the following: preoperatively, complete absence of blood flow signals in the left common femoral vein and popliteal vein (peak velocity 0 cm/s); on postoperative day 5, peak flow velocity in the left superficial femoral vein had recovered to 16 cm/s, with intermittent blood flow signals visible in the popliteal vein at approximately 13 cm/s; the femoral vein spectral waveform had returned to a phasic pattern fluctuating with respiration.

Following discharge, the patient was advised to undergo anticoagulation with rivaroxaban (15 mg twice daily for 21 days, then 20 mg once daily for 3 months) and to attend an outpatient ultrasound scan at 1 month to assess PTS. The 1-month follow-up was completed; the patient experienced no recurrence of edema. Subsequently, due to personal circumstances, the patient did not attend further follow-up.

## 3. Discussion

IVCS results from chronic compression of the left common iliac vein by the right common iliac artery and the L5 vertebral body, leading to endothelial injury, collagen deposition, and luminal stenosis.^[1,4]^ Although this anatomic variant is present in approximately 20% of the general population, symptomatic disease predominantly affects women aged 20 to 40 years.^[4]^ However, male patients typically present with acute, severe disease and are more likely to progress to PCD – a limb-threatening emergency with mortality rates approaching 40%.^[2,3,5]^ The present 54-year-old male lacked female-associated risk factors and progressed to PCD within 5 hours, exemplifying the acute, heavy thrombus-burden phenotype of male IVCS-DVT. This case underscores that IVCS is not exclusive to young women; suddenly, total left lower-limb DVT in middle-aged men should prompt immediate consideration of underlying IVCS.

Ultrasound serves 4 hierarchical roles in PCD emergency management. First, as a noninvasive bedside tool with diagnostic accuracy exceeding 95%,^[6]^ it rapidly locates thrombus and triggers time-critical intervention, in this case, directly enabling surgery within 2 hours. Second, after mapping DVT extent, it guides focused CTA assessment of iliac vein compression and directs venography, establishing an efficient stepwise pathway. Third, it aids differential diagnosis: here, absent foot pulses had been misdiagnosed as arterial thrombosis, but ultrasound revealed diminished distal arterial flow concurrent with full-length venous thrombosis, correctly attributing the absent pulses to venous hypertension-induced arterial spasm rather than primary arterial occlusion. Fourth, postoperative ultrasound quantifies recanalization; restored anterior tibial vein flow on day 5 (Fig. 2C) and femoral vein spectral recovery provided objective evidence of therapeutic efficacy.^[7,8]^

Despite its value, ultrasound carries significant limitations in identifying primary IVCS lesions. Deep pelvic location, bowel gas, and bony structures restrict the acoustic window; acute thrombus may obscure underlying stenosis, creating a false impression of primary thrombotic occlusion; and complete luminal occlusion can eliminate the expected focal velocity increase, producing a hemodynamic false-negative. In this patient, acute total occlusion from the external iliac vein distally masked the compressive lesion, rendering the iliac vein nonvisualized without indirect hemodynamic signs. Although clinicians should monitor for indirect signs – collateral circulation, markedly reduced proximal flow (<10 cm/s), and loss of respiratory phasicity^[4,9]^ – these are not consistently present. Therefore, for unexplained left lower-limb DVT, thrombus extending to the iliac level, or high clinical suspicion of IVCS, computed tomography venography, magnetic resonance venography, or venography should be completed within 48 hours, establishing an integrated pathway of ultrasound screening, cross-sectional confirmation, and intervention.

This case demonstrates that ultrasound is a pivotal component of the multimodal PCD pathway: it rapidly initiates emergency treatment, accurately assesses thrombus burden, and dynamically monitors recanalization. However, its limitations mandate a stratified imaging strategy with prompt computed tomography venography or magnetic resonance venography when IVCS is suspected.

## 4. Study limitations

This is a single-case report with inherent limitations. Several emergency-acquired ultrasound images were not archived. Partial intraoperative procedural log parameters, including original AngioJet pump operating settings and postoperative CDT infusion rates, could not be retrieved from emergency records. In addition, the patient was lost to follow-up beyond the 1-month outpatient visit, so definitive long-term PTS assessment and the need for secondary iliac vein stenting cannot be determined. Consequently, this study was only able to assess short-term clinical outcomes; it was unable to determine the incidence of PTS in the long term, nor could it objectively evaluate the necessity and long-term efficacy of secondary iliac vein stenting. The long-term prognosis of male patients with IVCS-PCD still requires further investigation based on a larger number of cases.

## Author contributions

**Conceptualization:** Ying Zhang.

**Data curation:** Weiwei Jiang, Ying Zhang.

**Formal analysis:** Ying Zhang.

**Funding acquisition:** Ying Zhang.

**Investigation:** Weiwei Jiang.

**Methodology:** Weiwei Jiang, Ying Zhang.

**Project administration:** Weiwei Jiang, Ying Zhang.

**Resources:** Ying Zhang.

**Software:** Ying Zhang.

**Supervision:** Ying Zhang.

**Validation:** Ying Zhang.

**Visualization:** Weiwei Jiang.

**Writing – original draft:** Weiwei Jiang.

**Writing – review & editing:** Ying Zhang.
